# Global extracellular volume (ECVglobal) in HCM - the "next generation" test for risk in hypertrophic cardiomyopathy?

**DOI:** 10.1186/1532-429X-15-S1-P140

**Published:** 2013-01-30

**Authors:** Daniel Sado, Viviana Maestrini, Steven K White, Stefan K Piechnik, Matthew D Robson, Andrew Flett, Maria T Tome Esteban, Antonios Pantazis, William J McKenna, Stefan Neubauer, Perry Elliott, James Moon

**Affiliations:** 1 The Heart Hospital, London, UK; 2 UCL, London, UK; 3 Oxford University, Oxford, UK

## Background

CMR LGE detects myocardial tissue abnormalities in hypertrophic cardiomyopathy (HCM) and aids in risk stratification, predicting in particular heart failure and to a lesser extent, sudden death. However, LGE quantifies only the area of focal fibrosis only, missing diffuse fibrosis and assuming all LGE is the same. We hypothesized that the total left ventricular fibrosis burden, as measured by the mean global myocardial extracellular volume (ECVglobal) using 16 segment T1 mapping, would be a superior marker.

## Methods

Fifty-six patients with HCM from a national cardiomyopathy centre were compared to 46 gender matched healthy volunteers. All participants underwent LGE imaging and ECVglobal quantification at 1.5T (Avanto, Siemens). LGE was quantified using the full width at half maximum technique. ECV quantification was performed using dynamic contrast equilibrium at 15 minutes following a single bolus of 0.1mmol/kg Dotarem. The T1 was assessed using the ShMOLLI sequence pre contrast and at dynamic equilibrium with hematocrit measurement on the same day. A 16 segment left ventricular model was used to derive the ECVglobal. In addition, LGE+ areas and LGE- segments were separately analyzed. All patients underwent full conventional HCM risk stratification and echocardiography.

## Results

**Figure 1:**
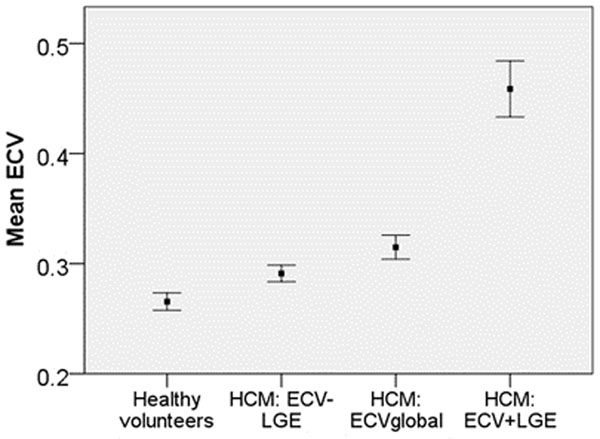


The ECVglobal was higher in HCM that in healthy volunteers, as was the ECV in LGE+ areas and LGE- segments. (0.32±0.04, 0.27±0.03, 0.47±0.01, 0.30±0.03, overall P<0.0001, Figure 1). The ECVglobal correlated with other markers of disease severity: systolic function (by CMR), diastolic function (by echocardiography), the occurrence of non sustained ventricular tachycardia, and the number of conventional risk factors for sudden death. When ECVglobal was compared to LGE extent for predictive power, ECVglobal was the better test.

## Conclusions

ECVglobal has potential to be a better CMR technique than LGE for tissue characterization in HCM.

## Funding

British Heart Foundation

**Table 1:** Pearson correlations and associations of the ECVglobal, ECV+LGE, ECV-LGE and %LGE

Variable	ECVglobal	ECV+LGE	ECV-LGE	% LGE
LV ESVi	r=0.35**	NS	NS	r=0.3*
EF	r=-0.35**	r=-0.3*	NS	r=-0.3
Risk factors for sudden death	↑**	NS	NS	NS
Non sustained ventricular tachycardia	↑**	↑*	NS	↑*
E:A ratio	r=-0.3*	NS	r=0.41**	NS
E;E'	r=0.36*	NS	NS	r=0.36*
%LGE	r=0.73***	NS	r=0.54***	N/A

*** = P<0.001 ** = P<0.001 * = P<0.05

